# Early acquisition of non-technical skills using a blended approach to simulation-based medical education

**DOI:** 10.1186/s41077-017-0045-2

**Published:** 2017-08-14

**Authors:** Andrew Coggins, Mihir Desai, Khanh Nguyen, Nathan Moore

**Affiliations:** 1 Emergency Department, Westmead Hospital, Hawkesbury Road, Sydney, NSW 2145 Australia; 2 Simulated Environment for Clinical Training (SiLECT), Sydney, Australia; 3 The University of Sydney, Western Clinical School, Sydney, Australia

## Abstract

**Background:**

Non-technical skills are emerging as an important component of postgraduate medical education. Between 2013 and 2016, a new blended training program incorporating non-technical skills was introduced at an Australian university affiliated hospital. Program participants were medical officers in years 1 and 2 of postgraduate training.

**Methods:**

An interdisciplinary faculty trained in simulation-based education led the program. The blended approach combined open access online resources with multiple opportunities to participate in simulation-based learning. The aim of the study was to examine the value of the program to the participants and the effects on the wider hospital system. The mixed methods evaluation included data from simulation centre records, hospital quality improvement data, and a post-hoc reflective survey of the enrolled participants (*n* = 68).

**Results:**

Over 30 months, 283 junior doctors were invited to participate in the program. Enrolment in a designated simulation-based course was completed by 169 doctors (59.7%). Supplementary revision sessions were made available to the cohort with a median weekly attendance of five participants. 56/68 (82.4%) of survey respondents reported increased confidence in managing deteriorating patients. During the period of implementation, the overall rate of hospital cardiac arrests declined by 42.3%. Future objectives requested by participants included training in graded assertiveness and neurological emergencies.

**Conclusions:**

Implementation of a non-technical skills program was achieved with limited simulation resources and was associated with observable improvements in clinical performance. The participants surveyed reported increased confidence in managing deteriorating patients, and the program introduction coincided with a significant reduction in the rate of in-hospital cardiac arrests.

## Introduction

Transitioning from undergraduate to postgraduate practice is a recognised challenge [1]. Junior doctors often experience high levels of anxiety and stress associated with the sudden increase in clinical responsibilities. A lack of preparedness in managing critical illness outside of normal working hours has been cited as a significant problem [2, 3]. In the Australian context, there is an increasing number of junior doctors working less hours in total. As a result, there is less real-life exposure to deteriorating patients and therefore fewer opportunities to acquire essential clinical skills [4].

‘Non-technical skills’ in healthcare include communication proficiency, decision-making, and teamwork [5, 6]. The term ‘non-technical’ is contentious because it may not fully emphasise the central importance of these skills for safe patient care [7]. Early acquisition of non-technical skills has been recognised as desirable in postgraduate training [8].

Non-technical skills can be acquired through both hands-on experience and through specific ‘Crisis Resource Management’ (CRM) training. The latter refers to the combination of teaching technical skills and non-technical skills with the recognition that both are essential components of safe patient care [9]. While learning ‘on the wards’ allows for acquisition over time, accelerated acquirement of these competencies in the initial stages of postgraduate training is preferable [10]. The use of a dedicated CRM training program in the early phase of a medical career has the potential to amplify learning from real-life clinical scenarios. Emerging evidence also supports the claim that early acquisition of these non-technical skills is beneficial [11, 12].

**Fig. 1:**
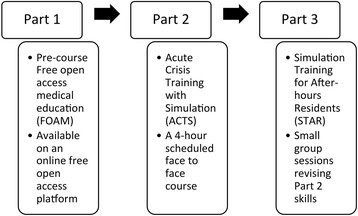
Non-technical skills training program

In 2013, our interdisciplinary faculty reviewed a series of local adverse events involving junior doctors attending Medical Emergency Teams (MET) calls. Specific analysis of these adverse events led by the local Advanced Life Support (ALS) committee suggested an association between clinical errors and suboptimal non-technical skills. As a result, it was postulated that specific training could be beneficial. The overall aim of the new program was to improve patient care by making sustainable improvements in meaningful areas such as handover and communication, while giving the doctors an approach to common MET call emergencies. Given the tension that exists between a junior doctor’s service provision and their need for training, it was determined that an innovative approach was required. After stakeholder consultation, we developed a targeted program (Fig. 1). The program combined open access online materials, a comprehensive CRM course, and brief follow-up sessions in order to consolidate acquired skills [13, 14].

The aim of this study was to explore participant’s experiences and examine the wider hospital effects in relation to the simulation program. From a local point of view, training in non-technical skills was being provided to a cohort of junior doctors for the first time.

## Method

### Setting and participants

The study setting was a tertiary centre affiliated with the University of Sydney in Australia. From 2012, Health Workforce Australia and the hospital executive provided resources to establish a local simulation centre (Simulated Environment for Clinical Training). The protocols for the study were examined and approved by the Western Sydney Local Health District (WSLHD) research and ethics committee.

For the purpose of this study, a ‘Junior Doctor’ was defined as a postgraduate year 1 ‘intern’ or postgraduate year 2 ‘resident’ starting their training between 2014 and 2016. Invited participants were junior doctors employed by the hospital on a 2-year contract. During pre-briefings, participants reported a variety of past exposure to simulation-based medical education in their undergraduate studies. Faculty were from both medical and nursing backgrounds and had specific skills in emergency medicine, anaesthesia, and critical care. In addition, hospital management and secretarial staff provided key support to the program.

### Non-technical skills program

The design of the program was divided into three distinct parts (Fig. 1) [13–15]. Part 1 consisted of pre-course reading materials that were presented on an open access online platform [15].

Part 2 consisted of a 4-h face-to-face course. The program objectives were divided into approximately $$ \raisebox{1ex}{$1$}\!\left/ \!\raisebox{-1ex}{$3$}\right. $$ intermediate life support (ILS) skills, $$ \raisebox{1ex}{$1$}\!\left/ \!\raisebox{-1ex}{$3$}\right. $$ matched simulation-based activities, and $$ \raisebox{1ex}{$1$}\!\left/ \!\raisebox{-1ex}{$3$}\right. $$ non-technical skills. The clinical content focused on important MET call presentations including anaphylaxis, respiratory failure, septic shock, and myocardial infarction. Simulation scenarios were delivered with either a faculty-simulated patient (anaphylaxis and septic shock) or adult manikin (cardiac arrest). A faculty confederate healthcare provider (registered nurse) was utilised for patient handover and participant reorientation if required. This approach was selected to achieve the best possible participant ‘immersion’ with the limited faculty available [16].

Part 3 consisted of scheduled revision sessions for participants who had enrolled in Part 1 and 2 of the program as well as other after-hours junior doctors that were on duty [15]. Part 3 was delivered in ‘protected teaching time’ at the start of after-hours shifts [15]. One hour was spent with four to eight invited participants. Advanced notifications were sent to the on duty after-hours doctors by phone text message using an existing hospital communications system. The 1 h was divided into a focussed 20-min skills session (themed as ‘*breathing’*, ‘circulation’, or ‘disability’) with a matched 40-min simulation activity. The content for Part 3 was derived from core content presented in Parts 1 and 2 of the program [15].

The target audience were doctors rostered on after-hours (afternoon) shifts. In the afternoon, there is a period of double staffing and therefore significant redundancy created by overlapping shifts. Faculty also reported that this time period (3.30 pm–4.30 pm) was favourable for them. While pragmatic, this approach to allocation led to unequal distribution in access across the cohort due to the variance in clinical rostering. As a result, a portion of enrolled doctors did not participate in all components of the program.

By using a relatively quiet portion of the doctors’ clinical commitment, Part 3 allowed for simulation-based learning without many of the typically associated costs [17]. The timing of the revision program was also advantageous as ideas discussed in the debrief (e.g. ISBAR handover) could be immediately applied in the clinical setting.

### Participant debriefing

Participant debriefing was undertaken by trained faculty from medical (*n* = 13) and nursing (*n* = 8) backgrounds. Training in simulation-based education included the completion of the National Health Education and Training in Simulation (NHET-Sim) course [18].

Following a 20-min skills station and 10-min simulation, a debrief was facilitated for 30 min. The terminal debriefs were not scripted. However, the debrief was structured into four phases (‘reactions’, ‘facts’, ‘analysis’, and ‘summary’). Time was equally allocated to clinical issues and non-technical skills [19]. The debrief was attended by two instructors with a ratio of faculty to participants of around 1:3. The use of video was not advantageous in this setting given the limited technical support available and small group sizes.

### Sampling and evaluation

**Fig. 2:**
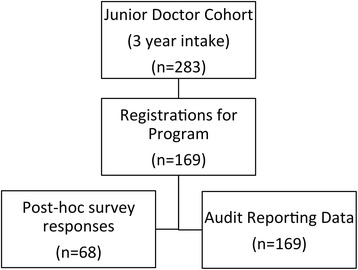
Educational program evaluation

The program was evaluated using a mixed method evaluation (Fig. 2). Simulation centre activity data was prospectively collected by a single investigator between 1 January 2014 and 30 June 2016 (30 months). Stata version 11 (Stata Inc., USA) was used for descriptive statistics. No comparative statistical tests were used due to likelihood of confounders.

Junior doctors (*n* = 169) were invited by e-mail to respond to an anonymous online survey about their experience of the program in April 2016. Sixteen questions (Table 1) were uploaded to *SurveyMonkey®* [20]. The 16 questions were derived from the simulation centre evaluation forms. In retrospect, our questionnaire could have been improved by using a standardised program evaluation resource validated by subject experts [21].

**Table 1:** Post-course participant survey

Number	Type	Survey question or statement
1	Yes, no	Have you been a junior doctor at Westmead Hospital in 2014, 2015 or 2016?
2	2014–2016	In what year were you an intern?
3	Number	How many terms have you completed at Westmead Hospital?
4	Likert scale	Overall, on a scale of 0–10 (10 = extremely useful and 0 = not at all useful) how has the overall experience of JMO “Simulation-based education” been at Westmead?
5	Yes, no	Other than the compulsory training during induction week, did you participate in any other simulation activities at Westmead Hospital?
6	Yes, no	(if *YES* to Question 5) Acute Crisis Training with Simulation (ACTS) for JMOs is a standalone 4-h course that has been running since 2014. Did you participate in an ‘ACTS’ program?
7	Likert scale	For this program rate your experience from 0 to 10 (10 = extremely useful and 0 = not at all useful)
8	Up to 3 statements	On reflection, looking back at the ‘ACTS’ program, what do you recall to be the MOST VALUABLE aspects of this course for your future practice?
9	Yes, no, N/A	Do you believe you are providing SAFER patient care as a result of this training?
10	More, less, about the same	As a result of the simulation programs you have experienced at Westmead Hospital: Do you feel more confident managing the “deteriorating (unstable) patient”?
11	Yes, no	Did you participate in after-hours “relief term” 3.30 pm ‘Simulation Training for After-hours RMOS’ (STAR) program?
12	Number	Approximately how many of these sessions did you attend?
13	Up to 3 statements	If you participated in the ‘STAR’ program: What were the 3 MOST valuable aspects of these sessions?
14	More, less, about the same	Do you believe you (or future junior doctors) would benefit from increased access to simulation, about the same amount or less simulation training?
15	Up to 3 statements	Name specific 3 issues that detract in anyway from your overall experience of simulation?
16	Up to 3 statements	Which scenarios commonly faced in your practice could be covered using simulation as an educational tool?

Survey questions were included to ascertain demographics and prior participation. Free-text entry was invited in order to describe the value of educational sessions especially in regard to timing and suitability. Free text was coded using conventional qualitative content analysis with the intent of identifying specific themes (Table 2). A single investigator collated responses electronically between April and July 2016.

**Table 2:** Participant survey—feedback (*n* = 68)

	Themes from qualitative content analysis of survey evaluation					
‘Most valued aspect of CRM training?’	Feedback	Practice	ECG	Learning environment	Communication	Blood gases
‘Most valued aspect of revision simulations?’	Teamwork	Deteriorating patient	Common scenarios	Debriefing	Calling for help	Communication
‘Issues that detracted from the experience?’	Manikin ‘not real’	Group ‘too large’ or ‘too small’	Pagers received during session	Debriefing was ‘too long’	Actors ‘distracting’	Timing of sessions before a busy shift
‘Which other scenarios would be useful for future learning?’	Haemorrhage (Gastrointestinal bleeding and trauma)	Neurological (Coma, delirium, and seizures)	Escalation and graded assertiveness	Respiratory (shortness of breath)	Septic shock (hypotension and fever)	Cardiac (bradycardia and tachycardia)

Data from the simulation centre records provided quantitative attendance numbers for auditing and reporting purposes. While these records were complete, a clear picture of an individual’s attendance pattern to Part 3 was not discernible because names were not recorded. A small proportion of responses from survey questions were incomplete due to omission (Table 3). The majority of omissions were viewed as appropriate as not all respondents had attended all parts of the program when surveyed.

**Table 3:** Participant survey evaluation (*n* = 68)

	Yes/more	No/less	No response	About the same
Do you believe you are providing safer patient care as a result of this training? n (%)	51/68 (75.0%)	2/68 (2.9%)	8/68 (11.8%)	7/68 (10.3%)
Do you feel more confident managing the deteriorating (unstable) patient? n (%)	56/68 (82.4%)	1/68 (1.5%)	2/68 (2.9%)	9/68 (13.2%)
Do you believe future interns would benefit from increased access to Simulation? n (%)	49/68 (72.1%)	0/68 (−)	2/68 (2.9%)	17/68 (25%)

## Results

Over the study period of 30 months, 283 junior doctors were invited to enrol in the new program. Completion of the 4-h training course (Part 2) was achieved by 169/283 (59.7%). The online course materials (Part 1) were accessed a total of 939 times [15]. The follow-up survey response rate was 68/169 (40.2%).

The faculty delivered 82 brief simulation-based revision sessions (Part 3) over the study period. Each revision session had a median attendance of five doctors (range 2–9). Overall, 48/68 (70.5%) of surveyed participants attended one or more of these sessions. Variance in rostering resulted in revision sessions being attended by rotating doctors who had not enrolled in the Part 2 course and some enrolled participants attending multiple sessions.

Overall experience of the program was rated ≥6/10 by 65/68 (95.6%) of survey respondents (Table 4). 56/68 (82.4%) felt more confident, and 51/68 (75.0%) stated they were providing safer care (Table 3). 49/68 (72.1%) of respondents stated access to simulation training should be increased, and 17/68 (25.0%) stated access to simulation should remain the same.

**Table 4:** Participant survey—overall experience (*n* = 68)

Overall rating of simulation experience	No answer	1 (not useful at all)	2–5	6	7	8	9	10 (extremely useful)	μ = 8.21 S.D. = 1.84
Number of responses n (%)	1 (1.5%)	2 (2.9%)	0 (−)	6 (8.8%)	12 (17.6%)	15 (22.1%)	12 (17.6%)	20 (29.4%)	

Content analysis of participant responses indicated that this cohort of learners would like further simulation training. A range of areas were suggested for future training including the management of haemorrhage, clinical escalation, cardiac emergencies, neurological deterioration, and sepsis (Table 2). Participants felt that detractors from their experience included the use of a manikin, pager interruptions, and group sizes (too large or too small).

Table 5 shows the local reporting of MET calls and cardiac arrests before and after the program in 2013 and 2016. A decline in cardiac arrest rates was observed (42.3%) with an increase in the rate of calling for help on the two-tiered MET call system.

**Table 5:** Reported cardiac arrests and Medical Emergency Team (MET) calls

Variable	Full year 2013	Full year 2014	Full year 2015	Full year 2016	% Change 2013–2016
Number of reported in-hospital cardiac arrests (overall total)	67	45	41	38	−42.3%
Number of MET Calls—Level 1 response (a primary team review)	6409	7017	8342	8696	+26.3%
Number of MET Calls—Level 2 (a full life support team)	1266	1473	1706	2037	+37.9%

## Discussion

A recent postgraduate training study concluded ‘there is a pressing need for medical schools and deaneries to review non-technical training to include more than communication skills’ [8]. In 2013, our faculty were dually involved in quality improvement and delivery of simulation. We noted an increase in adverse event reporting to the advanced life support committee. As a result, the management of deteriorating patients was flagged as a priority for medical education.

Our team concluded that a longitudinal approach to learning non-technical skills was required because there was concern that once-off simulations were unlikely to create the necessary impact on hospital-wide culture. The proposed non-technical skills program was to take place at a newly established simulation centre with no full-time staff. In view of the limited resources available, embedding non-technical skills within accessible training was a considerable challenge requiring an innovative approach. Prior to this program’s introduction, the only available training was aimed at senior staff using external simulation centres.

To meet the challenges described, the non-technical skills program was introduced in protected teaching time with support from hospital management. The described program was innovative in a number of respects including use of online free open access medical education, longitudinal use of simulation, opportunity for immediate application of skills, and an effective use of existing hospital resources including text messaging notifications.

The evaluation results show that participants engaged well with the program (Table 6). The majority surveyed reflected that they were providing safer care and would like the opportunity for more simulation training. Content analysis of free-text responses revealed a number of areas for future development (Table 2) which have since been incorporated in a 2017 revision of the program’s content.

**Table 6:** Overview of the blended CRM program

Variable	Full year 2014	Full year 2015	Half year 2016	Total
Intern intake (number of postgraduate year 1 doctors)	96	98	89	283
Part 1—course manual views online	85	489	365	939
Part 2—number of attendees at 4-h CRM program	36	73	60	169
Part 3—number of weekly revision sessions	20	36	26	82
Attendance of weekly revision simulations (median)	4	5	6	N/A
Total number of trained medical faculty	3	10	13	13
Total number of trained nursing faculty	4	6	8	8

From a local point of view, the main perceived benefit of this program was an initiation of culture change in our medical emergency teams. The importance of the relationship between team training and patient safety has been described in other settings (e.g. *TeamSTEPPS*™) [22]. In our institution, observed improvements following participation in the program included medical teams ‘calling for help early’ and a decline in the rate in-hospital cardiac arrests. The review of hospital reporting data (Table 5) shows a yearly decline in the cardiac arrest rate and a corresponding increase in the rate of calls to the two-tiered MET call system. We cannot conclude that the program was causal of this trend, but the association with meaningful improvements in outcomes is encouraging. Furthermore, survey responses (Table 2), feedback from senior staff regarding junior doctor performance, and observed improvements in clinical handover suggest the program has had a hospital-wide impact. An ideal postgraduate team training program should involve participants from various backgrounds working together. As a surrogate, we used trained faculty from a nursing background as confederate members of the simulation team and as debriefers [23].

Simulation-based education is applicable to many different disciplines and levels of experience [24, 25]. From a postgraduate training perspective, junior doctors often receive training in ALS skills on a ‘once off’ basis but usually have no further chance to consolidate their learning [26]. Attrition of skills acquired in simulation training was a key issue that we considered. We sought to prevent attrition with rostered revision sessions (Part 3). The Part 3 component was innovative in providing longitudinal opportunities for simulation with the aim of consolidating skills and sustaining lasting behavioural change. In other settings, this approach to training in combination with a refined MET call system has been shown to reduce hospital mortality [27].

In regard to costs, the simulation portion of the program was significantly strengthened by the use of ‘in kind’ resources and an interdisciplinary faculty (Table 6). From our experience, sustainable use of simulation-based training required accurate lesson planning, skilled faculty, and appropriate selection of simulation hardware. Furthermore, engagement with postgraduate managers is essential to ensure a consistent attendance. As a result of support from the postgraduate managers, this program did not require changes to junior doctor rostering or the simulation centre budget.

While the majority of participants stated that they would benefit from more simulation training (Table 3), our reported percentage is lower than similar contemporary studies [28]. The lower figures reported are for unknown reasons. A possible explanation would be participants’ exposure to simulation at an undergraduate level. Simulation exposure varies considerably between Australian medical schools, so some content may have been considered repetitive by a proportion of participants. In 2017, we modified the program for future cohorts to account for the feedback summarised in Table 2. Changes included redirecting doctors’ pagers, improved time-keeping, and an update of the clinical content [15]. The proportion of simulation-based content has remained unchanged to ensure the key learning objectives are fulfilled.

In terms of limitations, our survey response rate (40.2%) was lower than anticipated which may have led to non-response bias. The sub-optimal response rate was in part due to restrictions placed by the approving ethics committee in contacting participants (limited to an e-mail invitation from the postgraduate manager). While the approach of our program aimed to maximise long-term recall through regular revision, we did not assess participants with objective measures of their performance. From the results, we note that increased confidence following training is pleasing but that ‘self-rating’ following simulation should be considered a low-level outcome measure [29]. Furthermore, outcome measures of this type may not correlate with actual clinical competence [30].

## Conclusion

Acquisition and retention of non-technical skills is a current challenge in postgraduate medical education. Successful implementation of a new non-technical skills program was aided by support from hospital management and direct involvement of the faculty in quality improvement committees. In our experience, training for junior doctors was achievable even with limited simulation resources. Retention of new skills and culture change was supported by longitudinal opportunities for additional simulation. Program implementation coincided with a yearly decline in the hospital cardiac arrest rate and resulted in a self-reported increase in confidence by the participants.
